# Health emergency operation centers implementation challenges in Africa

**DOI:** 10.11604/pamj.2019.33.171.17890

**Published:** 2019-07-04

**Authors:** Abdoulaye Bousso

**Affiliations:** 1 Health Emergency Operation Center, Ministry of health of Senegal, Dakar, Senegal

**Keywords:** Health emergency, operation center, emergency

## Abstract

The African continent faced to many public health events. An effective organization is the key element for managing public health events. Health Emergency Operations Centers (HEOC) are strategic bodies that can help control such situations. We would like to share our experience about the HEOC implementation in Senegal, which is among the first in Africa while highlighting the challenges we have the HEOC concept is quite common in other continents, it is an innovation in Africa. And yet, Africa is the most affected by emergency situations and disasters and very few of its country possess HEOC. Consequently, emergency and disaster management interventions are done in a nonsystematic manner. The comprehension of the concept, the mission and organization need to be well understood for setting up a HEOC the added value of HEOC is great for African country to face public health events.

## Essay

The recent upsurge of public health events of international concern reveals how important it is for each country to create permanent surveillance, preparation and response mechanisms to ensure its internal health security and additionally contribute to global health security. The Ebola virus epidemic that has stricken few West Africa countries demonstrated their poor capacities to anticipate health disasters. There were almost no emergency coordination structures among these countries. Therefore, provided responses were unprepared without any anticipated coordination mechanisms to manage the full implementation of the emergency and prevention plans. Africa is affected every year by several public health events [1, 2] with significant risk of rapid spread due to the poor detection and response systems. This is the main rationale that explains the urge to create health emergency operation centers (HEOC). HEOCs have been growing in certain big nations with main purpose to manage public health emergencies and disasters [3]. Our governments also must change their paradigm and invest in emergency preparedness, [4-6] instead of waiting for a disaster to occur. An improvised disaster management may cause more expenses and can take a toll on our financial resources [2, 6, 7]. However, the creation of an HEOC require a long process that needs a solid understanding of the concept. We would like to share our experience in the creation of Senegal's HEOC, among the first in Africa.

### Understanding the concept of HEOC

The public health events affecting our continent are more likely predictable, due to recurrent occurrences in a very favorable geographical and environmental context. Most of our governments do not have official bodies totally dedicated to emergency management. Therefore, the disaster management is performed in a very uncoordinated manner. Interventions are split between various actors and response action plans are developed by different health sectors without any linkage between them. HEOC creations is has become a top priority in our countries, given the public health events striking us every year. Plus, the scarcity of our resources compels us to better rationalize our funds, therefore a coordination unit would facilitate this process. The HEOC provide not only response but also pre-crisis assistance (risk mapping, rapid response teams, training). The highest health and government authorities must understand this concept very well [8]. An analysis of the country emergency organization and management needs to be performed. In Senegal, not only the Ministry of Health operate during crisis, but also other ministerial entities such as the Ministry of the Interior and the Ministry of Armed Forces coordinate an effective administration of emergency and disaster assistance. An exhaustive understanding of the HEOC concept enables to easily identify the potential actors and position the HEOC. The first action to take when implementing an HEOC is to opt for a participative approach where all the relevant government stakeholders are fully involved. This will allow to eliminate reluctance from certain bodies of the MOH and any leadership concerns that would happen eventually.

The government stakeholders need to be explained and shown that the HEOC is neither competing for a starring role nor overshadowing them or snatching their responsibilities. They also need to be stipulated that the HEOC is a coordination and support unit that however must be officialized in a legal document signed by the entitled competent authority which is the Minister of Health [9]. The first raised concern during the Senegal HEOC creation was *"Do we really need an HEOC"*. To answer this, the strong argument we brought forward was the added of value of an HEOC, through the drafting of plans and procedures, the implementation of fully trained rapid response teams as well as an incident management system and the documentation of all public health events. All the above was not existing before the advent of HEOCs. Others needed to be clarified about the difference between public health emergencies and medical emergencies. Luckily, the personal involvement of the Ministry of Health helped remove any subjective bias. As the result, a successful HEOC implementation requires a Minister of Health endowed with a strong leadership. Another significant addressed concern was *"Could the Ministry of Health coordinate various sectors"*. The answer was positive. Despite all constraints, the Ebola epidemic response has been wholly run by the Ministry of Health. However, during the outbreak, the Ministry of Health had chaired a committee to fight the disease, in which were represented the Ministries of Finance, Armed Forces, Interior, Livestock and Communication.

### Defining the HEOC possible missions

The HEOC can bear two possible missions. It can either solely focus on epidemics; in other words, the center will only work on potentially epidemic disease management. Or it can focus on all emergency matters impacting public health. For instance, the center's missions will outlook epidemics and intervene in all emergency situations and disasters that impact populations' health statuses [3, 8]. Hence, an evolutive framework of the HEOC and its boundaries must be defined. A good identification of actors and other bodies involved in emergency management is a priority so that leadership concerns can be anticipated, and the most suitable option can be chosen for the country. Whichever option is taken, the HEOC must be able to intervene throughout the whole emergency management cycle: mitigation, preparedness, response and recovery [9]. Senegal's HEOC has opted for the second mission.

### What institutional anchorage?

This is a crucial question to address as it is noteworthy for the center's development. This will allow to define the relationship between the center, the Ministry of Health departments and the other governmental and non-governmental stakeholders. In most African countries, emergencies management are under the responsibility of the Ministry of Health and of the Interior. In other cases, they are under the control of the Prime Minister or the President. A great analysis of a country emergency management system must be conducted to define the best anchorage. Whichever the choice is, the HEOC is totally entitled to lead all the actors involved in health response. The autonomy of the HEOC to trigger and coordinate response depend on the entity in which it is anchored. Based on our experience, the legitimate HEOC anchorage is the Ministry of Health. Moreover, the anchorage can be done at the Minister's Office level or at the General Health Directorate level or in another Directorate level. Nevertheless, the HEOC must have the capacity to coordinate the entire health sectors involved in the response with procedures that define the collaboration rules and guidelines with the other sectors.

### What organization chart model?

The organization chart model depends on the HEOC's mission and the country health system architecture. Though there is a common basic organization for HEOCs, there is no standard model [3, 8, 10] which means that the existing models cannot be replicated. Senegal has learned from the different models used in similar countries and adapted their organization chart to their local context. Our chart has been dynamic as we initially started off with four units: operations, administration and finances, epidemiological surveillance and communication. The idea of a communication unit mainly came from the Ebola Epidemic outbreak. After the outbreak, we then turned it into a planification unit and appointed a communication manager that reports directly to the HEOC Director. Thus, the organization chart strictly goes by the HEOC's missions.

### What human resources?

Emergency and disaster management is a new field of study is our countries in terms of the Public health discipline. The HEOC staff must be trained according to the center's orientations and the country contextual realities. Specific knowledge is required in emergency management, action plans and procedures development, as well effective and efficient logistics and communication. Involved in a multidisciplinary and a multi-sectoral setting, HEOC staff must undergo a thorough training so that they can successfully coordinate all the different stages of emergency response and effectively accomplish their missions [11]. Task and responsibilities must be clearly defined [3, 8-10] HEOC staff also need to receive Academic (universities) and practical (international organizations) trainings, along with all the other partners who closely work with the center.

Population protection through a global health security plan absolutely start with the creation of an emergency management structure in each country. Though it has been existing in the developed world [11], it is rather uncommon in most African developing countries. The creation of HEOC is a must for our countries to rationalize our scarce resources and be able to promptly anticipate and manage emergencies and disasters.

## Competing interests

The author declares no competing interests.
